# A novel *Ruminococcus gnavus* clade enriched in inflammatory bowel disease patients

**DOI:** 10.1186/s13073-017-0490-5

**Published:** 2017-11-28

**Authors:** Andrew Brantley Hall, Moran Yassour, Jenny Sauk, Ashley Garner, Xiaofang Jiang, Timothy Arthur, Georgia K. Lagoudas, Tommi Vatanen, Nadine Fornelos, Robin Wilson, Madeline Bertha, Melissa Cohen, John Garber, Hamed Khalili, Dirk Gevers, Ashwin N. Ananthakrishnan, Subra Kugathasan, Eric S. Lander, Paul Blainey, Hera Vlamakis, Ramnik J. Xavier, Curtis Huttenhower

**Affiliations:** 1 Broad Institute of MIT and Harvard, Cambridge, MA 02142 USA; 2 Center for Computational and Integrative Biology, Massachusetts General Hospital and Harvard Medical School, Boston, MA 02114 USA; 3 Gastrointestinal Unit and Center for the Study of Inflammatory Bowel Disease, Massachusetts General Hospital and Harvard Medical School, Boston, MA 02114 USA; 4 Center for Microbiome Informatics and Therapeutics, Massachusetts Institute of Technology, Cambridge, MA 02139 USA; 5 MIT Department of Biological Engineering, Massachusetts Institute of Technology, Cambridge, MA 02142 USA; 6 Emory University School of Medicine, Emory University, Atlanta, GA 30322 USA; 7 MIT Department of Biology, Massachusetts Institute of Technology, Cambridge, MA 02142 USA; 8 Department of Systems Biology, Harvard Medical School, Boston, MA 02114 USA; 9 Department of Biostatistics, Harvard School of Public Health, Boston, MA 02115 USA; 10 Current address: Vatche and Tamar Manoukian Division of Digestive Disease, David Geffen School of Medicine at UCLA, Los Angeles, CA 90095 USA; 11 Current address: Janssen Human Microbiome Institute, Janssen Research & Development, Cambridge, MA 02142 USA

## Abstract

**Background:**

Inflammatory bowel disease (IBD) is characterized by chronic inflammation of the gastrointestinal tract that is associated with changes in the gut microbiome. Here, we sought to identify strain-specific functional correlates with IBD outcomes.

**Methods:**

We performed metagenomic sequencing of monthly stool samples from 20 IBD patients and 12 controls (266 total samples). These were taxonomically profiled with MetaPhlAn2 and functionally profiled using HUMAnN2. Differentially abundant species were identified using MaAsLin and strain-specific pangenome haplotypes were analyzed using PanPhlAn.

**Results:**

We found a significantly higher abundance in patients of facultative anaerobes that can tolerate the increased oxidative stress of the IBD gut. We also detected dramatic, yet transient, blooms of *Ruminococcus gnavus* in IBD patients, often co-occurring with increased disease activity. We identified two distinct clades of *R. gnavus* strains, one of which is enriched in IBD patients. To study functional differences between these two clades, we augmented the *R. gnavus* pangenome by sequencing nine isolates from IBD patients. We identified 199 IBD-specific, strain-specific genes involved in oxidative stress responses, adhesion, iron-acquisition, and mucus utilization, potentially conferring an adaptive advantage for this *R. gnavus* clade in the IBD gut.

**Conclusions:**

This study adds further evidence to the hypothesis that increased oxidative stress may be a major factor shaping the dysbiosis of the microbiome observed in IBD and suggests that *R. gnavus* may be an important member of the altered gut community in IBD.

**Electronic supplementary material:**

The online version of this article (doi:10.1186/s13073-017-0490-5) contains supplementary material, which is available to authorized users.

## Background

Inflammatory bowel disease (IBD) is a chronic inflammatory disease of gastrointestinal tract with two main clinical manifestations: Crohn’s disease (CD) and ulcerative colitis (UC). The pathogenesis of IBD in genetically susceptible individuals likely involves an overactive immune response to the gut microbiome [1]. Changes in the intestinal microenvironment may contribute to the altered gut microbial community composition in IBD. The inflammation of IBD is associated with increased generation of reactive oxygen species (ROS) and reactive nitrogen species (RNS), causing oxidative stress for both host cells and the gut microbiome [2–4]. Additionally, the gut microbiome in IBD shows dysbiosis characterized by increased abundance of functional pathways involved in the microbial response to oxidative stress [5]. However, the species and/or strains of microbes that contribute to this functionality remain unclear.

The increased availability of whole metagenome sequencing, as opposed to 16S rRNA gene sequencing, has provided the opportunity to study the taxonomic composition of microbiomes at unprecedented resolution, allowing strain-level and functional profiling. In addition, several computational tools for metagenomic sequencing data have recently enabled high-resolution study of specific strain variants within abundant species [6, 7]. It is increasingly clear that strain variability can result in important physiological and functional differences in how microbes interact with the host [8]. For example, different strains of the same species can provoke different host immune responses [8], and in the context of IBD, analysis of *Escherichia coli* showed a specific enrichment of adherent invasive *E. coli* (AIEC) strains in IBD vs. healthy individuals [9].

IBD is characterized by relapsing inflammation followed by periods of remission, highlighting the importance of tracking patients and their microbiome over time. Because the symptoms of IBD vary temporally, it is important to collect multiple samples from each patient to understand the changing landscape of the IBD gut microbiome. Furthermore, longitudinal studies of healthy individuals have revealed large inter-subject variations within the gut microbiome [10–12], suggesting the importance of understanding IBD disease-specific effects across multiple individuals. One notable study performed metagenomic sequencing on longitudinal IBD samples [13]. With recently developed tools, it is now possible to analyze large-scale, longitudinal, metagenomic cohorts to study the IBD gut microbiome at the strain level.

In this study, we aim to address two outstanding questions in IBD: 1) How does oxidative stress in IBD shape the composition of the gut microbiome at the species and strain levels? 2) Do IBD-associated strains have specific genomic adaptations that increase their fitness in the IBD gut? We used metagenomic sequencing of a longitudinal cohort to analyze functionally binned microbial species by their ability to tolerate oxidative stress, and we found increased abundance of facultative anaerobe species in the IBD gut. We detected dramatic, transient blooms in the relative abundance of *Ruminococcus gnavus* in IBD, often corresponding with increased disease activity. We further experimentally characterized aerotolerance of *R. gnavus* and identified an *R. gnavus* clade that is enriched in IBD patients and has a distinct functional repertoire including genes exclusively present in this clade. These genes often involve functions that may improve colonization of the IBD gut, including oxidative stress responses, adhesion, iron acquisition, and mucus utilization. This study highlights the importance of strain-level analysis to reveal IBD-specific taxonomic features and their functionality.

## Methods

### Sample collection

#### Samples from the PRISM study, collected at Massachusetts General Hospital

A subset of the PRISM cohort was selected for longitudinal analysis. A total of 15 IBD cases (nine CD, five UC, one indeterminate colitis) were enrolled in the longitudinal stool study (LSS). Three participants with gastrointestinal symptoms that tested negative for IBD were included as a control population. Enrollment in the study did not affect treatment. Stool samples were collected monthly, for up to 12 months. The first stool sample was taken after treatment had begun. Comprehensive clinical data for each of the participants was collected at each visit. At each collection, a subset of participants were interviewed to determine their disease activity index, the Harvey-Bradshaw index for CD participants and the simple clinical colitis activity index (SCCAI) for UC participants (Additional file 1: Table S1).

#### Samples collected at Emory University

To increase the number of participants in our analysis, a subset of the pediatric cohort STiNKi was selected for whole metagenome sequencing including five individuals with UC and nine healthy controls. All selected UC cases were categorized as non-responders to treatment [14]. Stool samples were collected approximately monthly for up to 10 months. The first sample from participants in the STiNKi cohort is before treatment started, and subsequent samples are after treatment started. Stool collection and DNA extraction methods are detailed in Shaw et al. [14].

### DNA and RNA isolation

#### Samples from the PRISM study, collected at Massachusetts General Hospital

DNA was extracted from stool using the QIAGEN AllPrep RNA/DNA Mini kit with an enzymatic and mechanical lysis step. Lysozyme and proteinase K were added to frozen stool as described in the QIAGEN RNAprotect Bacteria Reagent handbook with a 10-min incubation at room temperature while vortexing every 2 min. Samples were resuspended in RLT buffer and 0.1 mm glass beads were added for mechanical lysis with bead beating on a Mini Bead beater-8 from BioSpec products on the homogenize setting for 3 min. Debris was removed by two sequential centrifugation steps at maximum speed for 5 min. Supernatant was transferred to a QIAshredder spin column and homogenized lysate was added to the AllPrep spin column for DNA and RNA extraction using the QIAGEN protocol.

#### Samples collected at Emory University

Total nucleic acid was extracted via the Chemagic MSM I with the Chemagic DNA Blood Kit-96 from Perkin Elmer. This kit combines chemical and mechanical lysis with magnetic bead-based purification. Prior to extraction on the MSM-I, TE buffer, lysozyme, Proteinase K, and RLT buffer with beta-mercaptoethanol were added to each stool sample. The stool lysate solution was vortexed to mix. Samples were then placed on the MSM I unit. The following steps were automated on the MSM I. M-PVA Magnetic Beads were added to the stool lysate solution and vortexed to mix. The bead-bound total nucleic acid was then removed from solution via a 96-rod magnetic head and washed in three ethanol-based wash buffers. The beads were then washed in a final water wash buffer. Finally, the beads were dipped in elution buffer to re-suspend the DNA sample in solution. The beads were then removed from solution, leaving purified TNA eluate. The eluate was then split into two equal volumes, one meant for DNA and the other for RNA. SUPERase-IN solution was added to the DNA samples, the reaction was cleaned up using AMPure XP SPRI beads. DNase was added to the RNA samples, and the reaction was cleaned up using AMPure XP SPRI beads. DNA samples were quantified using a fluorescence-based PicoGreen assay. RNA samples were quantified using a fluorescence-based RiboGreen assay. RNA quality was assessed via smear analysis on the Caliper LabChip GX.

### Fecal calprotectin quantification

Fecal calprotectin (FCP) was assayed for each stool sample with the Eagle Biosciences Calprotectin Enzyme-Linked Immunoabsorbent Assay (ELISA) kit using standard protocols.

### Validation cohorts

Data from an IBD study by Lewis et al. [13] was downloaded from the NCBI SRA for use as an IBD validation cohort. Eighty samples from the Human Microbiome Project (HMP) were used as a healthy validation cohort [15].

### Analysis of whole metagenome sequencing data

The whole metagenome data were trimmed and human reads were filtered using KneadData using the default parameters. Community composition was calculated with MetaPhlan2 [16] using the default settings. Functional and pathway composition was calculated with HUMANn2 [17] using the UniRef90 database with default settings. Differentially abundant species were identified using MaAsLin. Strain-level analysis was performed using StrainPhlAn [7] with the default parameters and PanPhlAn [6] with (--min_coverage 1 --left_max 1.70 --right_min 0.30). While strain-level profiling using StrainPhlAn was carried out for 72 species of the gut microbiome with sufficient coverage, *R. gnavus* was the only species detected to have an IBD-specific strain.

The phylogenetic tree of *R. gnavus* strains was calculated from the *R. gnavus* marker gene SNP profiles, as produced by StrainPhlAn [7]. We used the phangorn R package to (1) calculate the distance between all profiles (using the Jukes and Cantor model); (2) construct the tree using a hierarchical clustering method (UPGMA (Unweighted Pair Group Method with Arithmetic Mean)); and (3) calculate the bootstrapped phylogeny using the bootstrap.pml function. To find species with decreased abundance during blooms of *R. gnavus*, we divided the samples into two groups: those with ≥ 10% relative abundance of *R. gnavus*, and those with < 10%relative abundance of *R. gnavus*. Then, we used MaAsLin to find differentially abundant species between the two groups.

### Binning by aerotolerance methods

Based on the relative abundance output of MetaPhlAn2 [16], we binned the species of the gut microbiome based on aerotolerance. We binned into two groups, anaerobes and facultative anaerobes. To define differential bins of aerotolerance, we started with the list assembled by [18] and expanded for abundant genera in our samples (Additional file 2: Table S2). Differences in the abundance of facultative anaerobes was calculated in R using nested ANOVA.

### Survival in aerobic conditions

*E. coli* Sakai (RIMD 0509952), *R. gnavus* (ATCC 29149), and *Eubacterium elegans* (ATCC 3376) were grown anaerobically at 37 °C in brain heart infusion media (BD) supplemented with 1% BBL vitamin K1-hemin solution (BD), 1% trace minerals solution (ATCC), 1% trace vitamins solution (ATCC), 5% fetal bovine serum (Hyclone), 1 g/L cellubiose, 1 g/L maltose, 1 g/L fructose, and 0.5 g/L cysteine. Confluent cultures diluted into 1 mL of fresh media and incubated anaerobically for 3 h at 37 °C, at which point dilutions of the cultures were plated to determine colony forming units (CFU). The cultures were then moved to an aerobic tissue culture rotator where they were incubated at 37 °C with loosened lids. Samples were taken anaerobically to determine CFUs after 1- and 3-h aerobic incubation.

### Sequencing of 11 *R. gnavus* strains

DNA libraries were constructed from 11 *R. gnavus* strains using a previously described automated microfluidic sample preparation device [19]. This device was used to minimize reagent cost and hands-on time. The device takes cells as input (<2 uL aliquots required) and subsequently performs enzymatic cell lysis, genomic DNA purification, tagmentation to construct DNA sequencing libraries, library cleanup, size selection, and elution. All mixing and elution steps are performed in the device, using valves in the two-layer microfluidic architecture. DNA capture and cleanup are achieved using solid phase reversible immobilization (SPRI) beads inside the device. The tagmentation reaction was performed following the Illumina Nextera protocol using the Tagment DNA Enzyme (Illumina), as described in Kim et al. [19].

The sequencing libraries from the device were barcoded with dual barcoding primers from the Broad Institute (Broad Genomics Platform). The entire volume of DNA library eluted from the device (8 μL) was mixed with 10 μL of NEBNext High Fidelity 2X PCR MasterMix, 1 μL forward primer, and 1 μL reverse primer. We performed PCR barcoding and amplification with the following protocol: 72 °C for 3 min; 98 °C for 30 s; 17 cycles of 98 °C for 10 s, 60 °C for 30 s, 72 °C for 30 s; and 72 °C for 5 minutes. The barcoded DNA was cleaned up with SPRI purification.

Barcoded libraries were quantified with Quant-iT (ThermoFisher) and pooled at equal concentrations. DNA sequencing was performed on Illlumina MiSeq (2 × 150 cycle runs).

### Assembly and annotation of new *R. gnavus* genomes

Raw MiSeq data were trimmed with Trimmomatic using the parameters: LEADING:3 TRAILING:3 SLIDINGWINDOW:4:15 MINLEN:80 Then, the data were assembled with SPAdes with the default parameters and annotated with Prodigal using the default parameters.

### Generation of a custom pangenome database for *R. gnavus*

Only three *R. gnavus* genomes were integrated into the default pangenome database supplied with PanPhlAn [6]. To create a more comprehensive pangenome database, we used an additional three reference genomes available from the NCBI as well as 11 newly assembled *R. gnavus* isolate genomes.

### Identification of IBD-specific genes

Using the coverage output for each gene in the pangenome from PanPhlAn [6], we identified IBD-specific genes, which are genes present in multiple IBD samples with greater than 10× cumulative coverage across all samples, that are undetectable in all controls from both the LSS and validation cohorts, including 80 stool metagenomes from the HMP. We used extremely stringent criteria for the identification of IBD-specific genes. Instead of only using samples where the coverage of *R. gnavus* is greater than 1×, as is the default for PanPhlAn, we used all samples. If a gene was present, even at very low coverage in a healthy sample, it was not considered to be an IBD-specific gene.

## Results

**Fig. 1:**
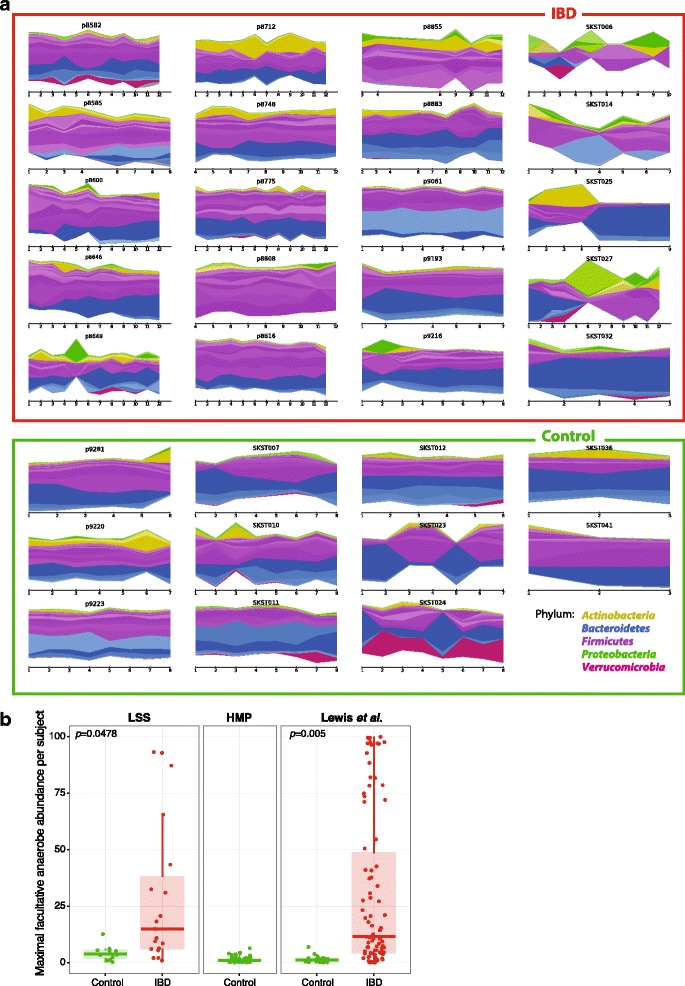
Dynamics of longitudinal microbial composition and facultative anaerobic microbial profiles in inflammatory bowel disease. **a** Individual microbial trajectories of 20 inflammatory bowel disease (IBD) patients and 11 control individuals with sufficient longitudinal data over time, from the current study (Massachusetts General Hospital, p-identifiers) and (Emory University, S-identifiers). Each subject exhibits an individualized microbiome signature. Each phylum has an overall color while genera are represented as different shades of the overall color. **b** Maximal relative abundances of facultative anaerobes across all subjects in the LSS (*n* = 266), Lewis et al. (n = 368) [13], and HMP (*n* = 80) [15] cohorts. Overall, facultative anaerobes are significantly higher in IBD patients compared to controls (nested ANOVA LSS *p* = 0.0478, Lewis et al. *p* = 0.005)

In order to determine how oxidative stress in IBD shapes the composition of the gut microbiome over time, we generated metagenomic sequencing data for a longitudinal stool study (LSS) including adult subjects from the PRISM (Prospective Registry in IBD Study at Massachusetts General Hospital) cohort as well as a pediatric cohort from Emory University which previously has been analyzed using 16S rRNA gene sequencing [14]. In total, the LSS cohort includes 266 samples from 20 IBD patients and 12 controls. Stool samples were collected monthly, for up to 12 months per patient, where the first stool sample was taken after treatment had begun. To validate and support our findings with other IBD cohorts, we additionally analyzed 331 metagenomics samples from Lewis et al. [13], a longitudinal microbiome study of CD patients with four time points, 0, 1, 4, and 8 weeks. To increase our analysis of healthy controls, we also examined 80 metagenomic samples from the Human Microbiome Project (HMP) [15]. An overview of these subjects’ phylum-level gut microbiome profiles stratified by genus gut microbial profiles shows the trajectory over time (Fig. 1a).

### Aerotolerance signature found in IBD differentially abundant species

To determine how the increased oxidative stress of the IBD gut shapes the composition of the gut microbiome, we first needed to bin the species of the gut microbiome by their ability to respond to oxidative stress (rather than by their taxonomic classification). Based on literature review (“Methods”), we manually binned species into two categories: i) oxygen-utilizing organisms (also known as facultative anaerobes, which can grow with or without oxygen); and ii) organisms that cannot use oxygen as a terminal electron acceptor (namely, aerotolerant and obligate anaerobes, which are both unable to grow in oxygen; however, unlike obligate anaerobes, aerotolerant bacteria are not rapidly poisoned by atmospheric oxygen) (Additional file 2: Table S2).

We found a consistent signal of increased facultative anaerobe abundance in IBD patients across both the LSS and the Lewis et al. cohorts (*p* value = 0.0478 and 0.005, respectively; Fig. 1b). IBD patients from the LSS and Lewis et al. cohorts showed mean relative abundances of facultative anaerobes of 9.73 and 19.48%, respectively. In contrast, control individuals from the HMP, LSS, and Lewis et al. cohorts showed mean relative abundances of facultative anaerobes of 1.36, 1.78, and 1.45%, respectively (Fig. 1b). Thus, IBD patients across three different clinical centers all showed a markedly increased abundance of facultative anaerobes. These facultative anaerobe species may have a fitness advantage compared to the other anaerobic commensals of the healthy gut, because they may be able to tolerate the increased oxidative stress of the IBD gut.

In addition to the overall class of facultative anaerobes that were enriched in IBD patients, we identified individual taxa that were significantly enriched or depleted (“Methods”). This detected 15 enriched and nine depleted species (q < 0.25, Additional file 3: Table S3). These agreed broadly with previous studies of gut microbial profiles in IBD, including depletion of *Clostridales* in *Bacteroidales* species (e.g., *Faecalibacterium prausnitzii*) as also observed in, e.g., Lewis et al. [13]. Previous studies have also observed increases of *E. coli*, *Klebsiella pneumoniae*, and *Veillonella* species in IBD patients [13]. While we observed blooms of these species in a few patients with IBD trending in the same direction, these did not reach statistical significance in our cohort and test. Other differences included a lack of *Bifidobacterium* species in our study; both of these discrepancies with respect to the Gevers et al. [20] cohort may be due to a combination of age (20–66 years here, 3–17 years in Gevers et al.) and sample type (stool vs. predominantly biopsy) differences between the studies.

Of the nine-identified species that showed consistent differences in abundance between IBD patients and controls in both the LSS and the Lewis et al. cohorts, eight could be explained by their tolerance to oxidative stress. Two facultative anaerobes showed increased abundance in IBD patients (*Streptococcus salivarius* and *Streptococcus parasanguinis*), whereas six anaerobes showed decreased abundance in the IBD samples (including *Blautia obeum* and *Alistipes putredinis*; Additional file 3: Table S3). These findings are consistent with previous observations of enriched abundance of proteobacteria species in the IBD gut, as these species are facultative anaerobes. The one remaining differentially abundant species, which was not apparently explained by its tolerance to oxidative stress, was *R. gnavus.* Despite being classified as an anaerobe, *R. gnavus* was more abundant in IBD patients. Indeed, of all bacterial species, *R. gnavus* showed the greatest increased abundance in IBD patients compared to controls in both the LSS and Lewis et al. cohorts (Additional file 3: Table S3).

### R. gnavus transiently dominates the gut microbiome of IBD patients

**Fig. 2:**
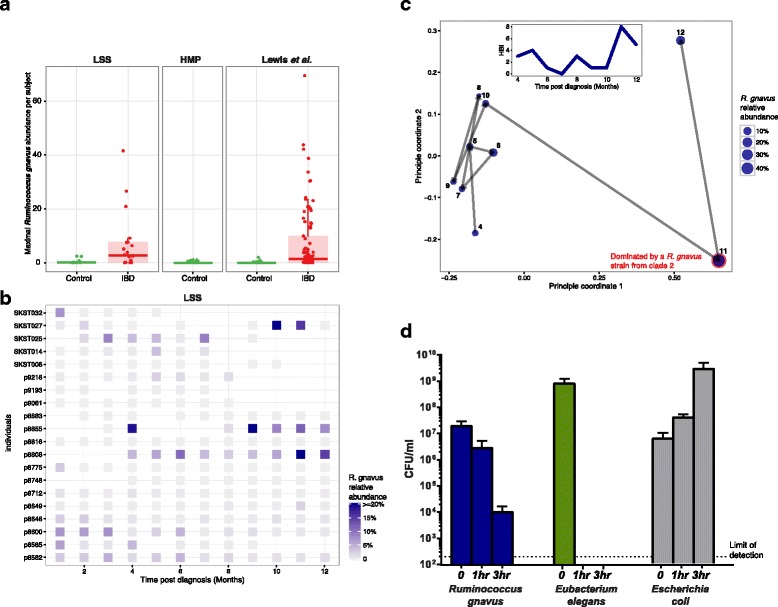
*R. gnavus* transiently dominates the gut microbiome in IBD. **a** The maximum relative abundance of *R. gnavus* across samples (time courses) is shown for all subjects in the LSS (*n* = 266), Lewis et al. (*n* = 368), and HMP (*n* = 80) cohorts. While the abundance of most anaerobes are lower in IBD patients, the abundance of *R. gnavus* is significantly higher in IBD patients compared to controls. **b** Relative abundance of *R. gnavus* over time for IBD patients in the LSS cohort. The abundance of *R. gnavus* is not constantly high, but rather has transient increases in the IBD gut. **c** A principle coordinate analysis (PCoA) of the Bray-Curtis distance of species-level microbial communities of LSS IBD patient p8808 over 9 months. The dominant *R. gnavus* strain in months 4–10 and month 12 is *R. gnavus* clade 1, while in month 11 the dominant strain of *R. gnavus* is *R. gnavus* clade 2 (Fig. 3). Inset shows the Harvey-Bradshaw Index (HBI) score, a clinical indication of active disease and inflammation, for this patient over time. The dramatic, transient increased abundances of *R. gnavus* in month 11 corresponds to an increase in HBI values (i.e., disease activity). **d** Colony forming units of *R. gnavus*, *Eubacterium elegans*, and *E. coli* at 0, 1, and 3 h post-transfer to atmospheric oxygen conditions (see “Methods”). *Error bars* represent standard deviation. *Dotted line* signifies limit of detection. No colonies were detected for the obligate anaerobe *E. elegans* at the 1- and 3-h time points. As expected, *E. coli* showed growth during oxygen exposure, and interestingly, despite being classified as an obligate anaerobe, *R. gnavus* was able to tolerate atmospheric oxygen for several hours, which may partially explain its increased abundance in the increased oxidative stress of the IBD gut

We identified *R. gnavus* blooms in 8/20 and 26/86 individuals from the LSS and Lewis et al. cohorts, respectively (defining a bloom as greater than 5% relative abundance). *R. gnavus* clearly stands out among all differentially abundant species as dominating the gut microbiome of IBD patients. The maximum relative abundance of *R. gnavus* in IBD patients from the LSS and Lewis et al. cohorts was 41.6 and 69.5%, respectively, whereas the maximum relative abundance was only 2.44% across all controls from these cohorts and only 1.06% in healthy HMP participants (Fig. 2a).

Interestingly, the increased abundances of *R. gnavus* in IBD patients were transient—typically spanning only one or two time points (Fig. 2b). These transient increases were detected in patients from the three different clinical centers and are therefore unlikely to be a geographical anomaly. In subject 8808 from the LSS cohort, a bloom in *R. gnavus* corresponded with a peak in the Harvey-Bradshaw Index (HBI), a clinical measure of disease activity, but the overall correlation between HBI did not reach significance (Fig. 2c). Blooms of *R. gnavus* also corresponded with significant decreases of previously identified IBD-depleted species, including *F. prausnitzii* (q-value = 3.04e-10; full list in Additional file 4: Table S4). The longitudinal nature of our cohorts was critical for this discovery, as it is impossible to identify transient blooms from cross-sectional data.

### Aerotolerance classification of *R. gnavus*

The increased abundance of *R. gnavus* was surprising in light of its classification as an obligate anaerobe because the other species increased in the IBD gut were facultative anaerobes. Therefore, we sought to test the bacteria’s ability to survive or grow in the presence of oxygen in the lab. We tested the aerotolerance of *R. gnavus* ATCC 29149 (isolated from healthy human stool [21]) relative to a facultative anaerobe (*E. coli*) and a strict anaerobe that was considerably less abundant in IBD (*E. elegans*). We found that while it is unable to grow in the presence of oxygen, *R. gnavus* retained 10^6^ viable cells after 1 h of atmospheric oxygen exposure and 10^4^ viable cells after 3 h in the presence of oxygen (see “Methods”). In contrast, *E. elegans* was unable to tolerate atmospheric oxygen at all, falling below our limit of detection even after only 1 h of exposure. The increased tolerance of *R. gnavus* to atmospheric oxygen may contribute to its blooms in the IBD gut (Fig. 2d).

### *R. gnavus* strains represent two phylogenetically distinct clades

The presence of *R. gnavus* in healthy and control samples, albeit in a much lower abundance, made us consider whether it had different functionality when present in high abundance in IBD patients. Sequencing many strains of bacteria from the same species has revealed that different strains can have dramatically different functionality due to a large number of highly variable accessory genes.

**Fig. 3:**
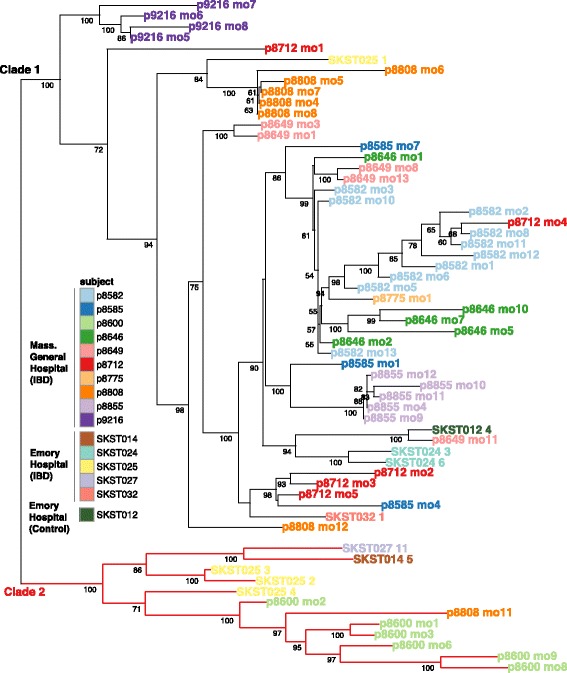
*R. gnavus* metagenomic strain phylogeny. A phylogenetic tree of *R. gnavus* strains, calculated from SNP profiles of *R. gnavus* marker genes (see “Methods”), where each tree leaf is a sample from the LSS or Lewis et al. cohorts (subject plus time point). Subject SKST012 is the only included control individual, as no other control metagenomes contained enough *R. gnavus* reads for detection and strain assignment by StrainPhlAn. Bootstrap values are indicated on branches and reveal two distinct clades of *R. gnavus* strains

First, we examined whether we could detect different strains of *R. gnavus* in our samples. One way to identify multiple strains would be to take a closer look at genes that are annotated as belonging only to *R. gnavus* genome (denoted “marker genes”). Nucleotide variations in reads mapping to such marker genes would suggest the presence of multiple strains of this species. Indeed, using single nucleotide polymorphism (SNP) profiles of *R. gnavus* marker genes, we identified two prominent clades of *R. gnavus* strains, which we named clade 1 and clade 2 (Fig. 3).

Most commonly, strains from the same individual belong to the same clade, with several exceptions. One notable case is patient 8808, whose gut microbiome underwent a shift between months 10 and 11, coinciding with an increase in the Harvey-Bradshaw Index (HBI) of disease. The higher abundance of *R. gnavus* found at month 11 (41.6% relative abundance) is not merely an expansion of the previous strain, belonging to clade 1, but rather a bloom of *R. gnavus* clade 2 (Figs. 2c and 3).

Second, we wanted to examine the functional differences between these two clades. We used the collection of gene families from all sequenced reference *R. gnavus* genomes (denoted as the *R. gnavus* pangenome) to search for genes that are differentially present in the two clades, suggesting different functional potential. To perform this analysis, we used the PanPhlAn tool [6]. Because its pre-computed pangenome database for *R. gnavus* lacked sufficient representatives from clade 2, we increased our pangenome coverage for clade 2 strains by sequencing and assembling genomes of 11 additional isolates of *R. gnavus* from IBD patients and infants (in addition to the currently existing six reference genomes; Additional file 5: Table S5). The new *R. gnavus* pangenome contained 11,933 genes with an average of 3117 genes per genome. Only 1178 genes were found in all 17 *R. gnavus* genomes, indicating that 74% of the *R. gnavus* genome is variable between strains. The size of the variable genome ranges from 1708 to 2473 genes.

The pangenome analysis on the 17 *R. gnavus* reference genomes confirmed the presence of two distinct clades as identified by their SNP profiles (Additional file 6: Figure S1). Two previously reported *R. gnavus* reference genomes were present in clade 2, but neither were from healthy adults (Additional file 5: Table S5). Using all metagenomic samples from the LSS, Lewis et al., and HMP cohorts, we found that *R. gnavus* strains from all controls and some IBD patients belonged to clade 1. On the other hand, only *R. gnavus* strains from IBD patients belonged to clade 2. Importantly, strains from clade 2 were present in patients originating from three different clinical centers in the US, suggesting the generality of this observation. The functional characterization of both clades highlighted three more cases of blooms of *R. gnavus* clade 2 (patients 9216, 8712, and 9193). Interestingly, *R. gnavus* clade 2 was also identified in infants from the DIABIMMUNE cohort (RJX1118 and RJX1119; Additional file 6: Figure S1) [10] where the abundance of *R. gnavus* is considerably higher than in healthy adults. Using 80 healthy controls from the HMP, no individuals with *R. gnavus* clade 2 were identified. Taken together, we concluded that *R. gnavus* clade 2 was enriched in strains from adults with IBD.

### *R. gnavus* clade 2 strains harbor IBD-specific genes

**Fig. 4:**
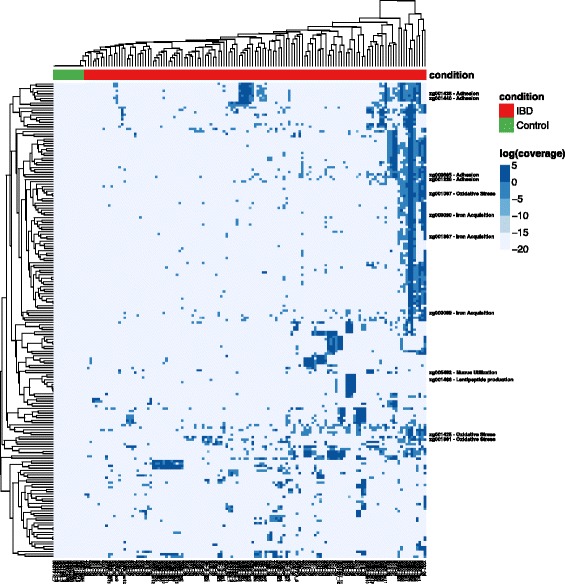
Functional profiles of IBD-related *R. gnavus* strains; 199 IBD-specific genes within *R. gnavus* (*rows*) and their depth of coverage across metagenomic samples (*columns*). All samples with at least 1× coverage of the *R. gnavus* pangenome are shown. Cluster of genes indicate that different strain groups (clades; Fig. 3) have different subsets of IBD-specific genes. Several gene families of interest are highlighted (see text), and the full *R. gnavus* pangenome can be found in Additional file 5: Figure S1

To understand the functional potential of each clade, we searched for individual *R. gnavus* genes that were not detected in any healthy individuals and present in at least two IBD samples with greater than 10× cumulative coverage. We identified 199 such IBD-specific genes (Fig. 4). These genes are involved in various functions that may improve colonization of the IBD gut, including oxidative stress responses, adhesion, iron acquisition, and mucus utilization, as well as a range of other general functions (Additional file 7: Table S6).

Three IBD-specific gene families are likely involved in the response to oxidative stress. Two IBD-specific genes encode peroxiredoxin and NADH oxidase, proteins that may act as a potent peroxidase, which could provide extra protection against oxidative stress in the IBD gut [22]. Another IBD-specific gene product, cystathionine gamma-lyase, is involved in the biosynthesis of cysteine, which is necessary for the biosynthesis of glutathione, an important antioxidant [23]. These results indicate that *R. gnavus* clade 2 strains may be specifically adapted to tolerate the higher oxidative stress of the IBD gut.

Three additional IBD-specific gene families are involved in the acquisition of iron. Iron is an essential nutrient for almost all bacteria, and is often a limiting nutrient. Additional genes involved in iron acquisition could be adaptive for colonization of the IBD gut because anemia is common in patients with IBD [24]. Two of these gene families were annotated as iron ABC transporters and the third as an enterochelin esterase, which makes iron chelated by siderophores available for use.

Previous studies have reported that some strains of *R. gnavus* utilize mucosal glycans. We found an IBD-specific gene involved in sialic acid transport, which may improve the efficiency of mucus utilization [25]. Thirteen IBD-specific gene families are involved in sugar utilization, including glycoside hydrolases and sugar transporters, which may allow for utilization of an expanded repertoire of sugars as carbon sources (Additional file 8: Table S7).

Finally, one IBD-specific gene is annotated as a lantipeptide synthetase which is involved in the biosynthesis of modified peptides, many of which have antimicrobial effects. Some strains of *R. gnavus* are known to produce the lantipeptide ruminococcin A, which inhibits the colonization of *Clostridium* species [26]. Lantipeptides could thus provide *R. gnavus* a competitive advantage in colonizing the gut.

The IBD-specific genes are likely evidence for collective adaption to the IBD gut by *R. gnavus* strains. The fact that nine of the genes are associated with mobile elements such as transposase, phage tail tape measure, or relaxosome proteins indicates that many of these genes were likely “cargo genes” of mobile genetic elements acquired by *R. gnavus* (Additional file 8: Table S7). Thus, the IBD-specific genes are not “core” genes for clade 2, but likely have been acquired and retained because they collectively provide an adaptive advantage in the microenvironment of the IBD gut.

## Discussion

The results presented here are concordant with the hypothesis that a major feature of the dysbiosis of the gut microbiome in IBD is a shift towards organisms that can cope with increased oxidative stress [27]. Importantly, resistance to oxidative stress is widely, yet sparsely, distributed throughout the gut microbiome phylogeny. It is thus not well addressed by simple taxonomic binning. The microbial shift towards facultative anaerobes has several important implications for the pathophysiology of IBD. We found that the strongest, yet transient, deviation in the microbial composition of IBD patients was the bloom of *R. gnavus* from an average of 0.1% in our controls to up to 69% in IBD patients with active disease. The physiologic niche of *R. gnavus* is likely mucolytic (utilizing glycans from the intestinal mucus layer as energy sources). We speculate that the dramatic change in *R. gnavus* abundance affects the delicate equilibrium of the mucus layer, due to the dramatic increase in the fraction of the community utilizing the mucus layer. This disequilibrium could in turn influence the gut barrier integrity, potentially increasing the intestinal permeability of IBD patients.

Whether the increased abundance of *R. gnavus* in IBD is a cause or effect of inflammation is currently unknown. At the very least, *R. gnavus* is a superlative colonizer of the inflamed IBD gut. However, it is also possible that *R. gnavus* contributes to or exacerbates the excessive immune response to the gut microbiome in IBD patients. In addition to its increased abundance in IBD, other studies have implicated *R. gnavus* as a potential pathobiont. Three cases of *R. gnavus* bacteremia and a case of infection of the bone surrounding a hip implant by *R. gnavus* have been reported [28–30]. In a study about treating UC patients with fecal microbiome transplants, patients who received stool from donors with high *R. gnavus* were more likely to relapse [31], suggesting a causal role in disease. Denser sampling of patients prior to flares, during their treatment, and after remission will enable the field to further examine the role of *R. gnavus* in IBD disease activity.

The original misclassification of *R. gnavus*, combined with 16S amplicon sequencing limitations for species-level identification, have confounded analysis in previous IBD studies, and left the significance of *R. gnavus* in the dysbiosis of IBD underappreciated. *R. gnavus*, a member of the phylum *Firmicutes*, is present in the gut of ~ 90% individuals (though at low abundance). Yet its genus classification has been recently disputed. Originally classified as a member of the *Ruminococcus* genus by Moore et al. [21], it has been recently transferred to the *Blautia* genus based on 16S rRNA gene sequencing [32, 33]. Specifically, true *Ruminococcus* species have decreased abundance in IBD patients, masking the increased abundance of *R. gnavus* in these samples, when examined only at the genus level.

## Conclusions

One major advantage of this study is that the coupling of longitudinal samples with whole metagenome sequencing has allowed us to study the IBD gut microbiome at the unprecedented resolution of strains and their functional potential. The accurate species- and strain-level identification afforded by whole metagenome sequencing is especially critical in understanding the abundance of *R. gnavus*, as 16S rRNA gene sequences from *Blautia* species are highly similar and most 16S analysis has therefore summarized at genus *Blautia*. Specifically, in IBD, the abundance of different *Blautia* species change in the opposite direction, completely masking *R. gnavus* blooms. We used the metagenomic sequencing to go beyond taxonomic profiling and study the functional differences among various *R. gnavus* strains. Taken together, our results reveal a clade of *R. gnavus* strains that may be better adapted to the IBD gut, through mechanisms of oxidative stress responses, adhesion, iron acquisition, and mucus utilization.

## Additional files

Additional file 1: Table S1. Clinical metadata for samples collected at Massachusetts General Hospital. (XLSX 14 kb)

Additional file 2 Table S2. List of genera binned by aerotolerance. (XLSX 9 kb)

Additional file 3. Table S3. Differentially abundant species in IBD. Differentially abundant species in IBD patients compared to controls that are significant in both the LSS and Lewis et al. cohorts (q < 0.20, using MaAsLin). (XLSX 14 kb)

Additional file 4: Table S4. Differentially abundant species during *R. gnavus* blooms. (XLSX 13 kb)

Additional file 5: Table S5. Isolation sources for all currently available R. gnavus genomes. Isolate genomes sequenced and assembled in this manuscript are indicated with an asterisk. (XLSX 9 kb)

Additional file 6: Figure S1. Pangenome analysis on two distinct clades of *R. gnavus*. Pangenome analysis of *R. gnavus* using PanPhlAn showing the presence or absence of every gene in the *R. gnavus* pangenome. The x-axis shows metagenomic samples as well as *R. gnavus* reference genomes; the y-axis shows gene clusters from the *R. gnavus* pangenome. Clustering the results reveals two clades of *R. gnavus*, which we call clade 1 and clade 2. Using metagenomic samples from LSS/Lewis/HMP cohorts, we found that *R. gnavus* strains from all healthy adult controls and some IBD samples were functionally similar to *R. gnavus* clade 1. On the other hand, only IBD samples had *R. gnavus* strains which were functionally similar to *R. gnavus* clade 2. The *red transparent rectangle* contains genes enriched in *R. gnavus* group IBD. The *green transparent rectangle* contains genes many genes missing from *R. gnavus* group IBD. (PDF 2396 kb)

Additional file 7: Table S6. Gene annotations for the selected *R. gnavus* IBD-specific genes mentioned in the main text. (XLSX 9 kb)

Additional file 8: Table S7. Gene annotations of all 199 IBD-specific genes. (XLSX 15 kb)

## References

[1] Khor B, Gardet A, Xavier RJ (2011). Genetics and pathogenesis of inflammatory bowel disease. Nature.

[2] Balmus IM, Ciobica A, Trifan A, Stanciu C (2016). The implications of oxidative stress and antioxidant therapies in inflammatory bowel disease: clinical aspects and animal models. Saudi J Gastroenterol.

[3] Poulsen NA, Andersen V, Møller JC, Møller HS, Jessen F, Purup S (2012). Comparative analysis of inflamed and non-inflamed colon biopsies reveals strong proteomic inflammation profile in patients with ulcerative colitis. BMC Gastroenterol.

[4] Seril DN, Liao J, Yang G-Y, Yang CS (2003). Oxidative stress and ulcerative colitis-associated carcinogenesis: studies in humans and animal models. Carcinogenesis.

[5] Morgan XC, Tickle TL, Sokol H, Gevers D, Devaney KL, Ward DV (2012). Dysfunction of the intestinal microbiome in inflammatory bowel disease and treatment. Genome Biol.

[6] Scholz M, Ward DV, Pasolli E, Tolio T, Zolfo M, Asnicar F (2016). Strain-level microbial epidemiology and population genomics from shotgun metagenomics. Nat Methods.

[7] Truong DT, Tett A, Pasolli E, Huttenhower C, Segata N (2017). Microbial strain-level population structure and genetic diversity from metagenomes. Genome Res.

[8] Geva-Zatorsky N, Sefik E, Kua L, Pasman L, Tan TG, Ortiz-Lopez A (2017). Mining the human gut microbiota for immunomodulatory organisms. Cell.

[9] Martinez-Medina M, Garcia-Gil LJ (2014). Escherichia coli in chronic inflammatory bowel diseases: An update on adherent invasive Escherichia coli pathogenicity. World J Gastrointest Pathophysiol.

[10] Yassour M, Vatanen T, Siljander H, Hämäläinen A-M, Härkönen T, Ryhänen SJ (2016). Natural history of the infant gut microbiome and impact of antibiotic treatment on bacterial strain diversity and stability. Sci Transl Med.

[11] Faith JJ, Guruge JL, Charbonneau M, Subramanian S, Seedorf H, Goodman AL (2013). The long-term stability of the human gut microbiota. Science.

[12] Franzosa EA, Huang K, Meadow JF, Gevers D, Lemon KP, Bohannan BJM (2015). Identifying personal microbiomes using metagenomic codes. Proc Natl Acad Sci U S A.

[13] Lewis JD, Chen EZ, Baldassano RN, Otley AR, Griffiths AM, Lee D (2015). Inflammation, antibiotics, and diet as environmental stressors of the gut microbiome in pediatric Crohn’s disease. Cell Host Microbe.

[14] Shaw KA, Bertha M, Hofmekler T, Chopra P, Vatanen T, Srivatsa A (2016). Dysbiosis, inflammation, and response to treatment: a longitudinal study of pediatric subjects with newly diagnosed inflammatory bowel disease. Genome Med.

[15] Human Microbiome Project Consortium (2012). Structure, function and diversity of the healthy human microbiome. Nature.

[16] Truong DT, Franzosa EA, Tickle TL, Scholz M, Weingart G, Pasolli E (2015). MetaPhlAn2 for enhanced metagenomic taxonomic profiling. Nat Methods.

[17] Abubucker S, Segata N, Goll J, Schubert AM, Izard J, Cantarel BL (2012). Metabolic reconstruction for metagenomic data and its application to the human microbiome. PLoS Comput Biol.

[18] Albenberg L, Esipova TV, Judge CP, Bittinger K, Chen J, Laughlin A (2014). Correlation between intraluminal oxygen gradient and radial partitioning of intestinal microbiota. Gastroenterology.

[19] Kim S, De Jonghe J, Kulesa AB, Feldman D, Vatanen T, Bhattacharyya RP (2017). High-throughput automated microfluidic sample preparation for accurate microbial genomics. Nat Commun.

[20] Gevers D, Kugathasan S, Denson LA, Vázquez-Baeza Y, Van Treuren W, Ren B (2014). The treatment-naive microbiome in new-onset Crohn’s disease. Cell Host Microbe.

[21] Moore WEC, Johnson JL, Holdeman LV (1976). Emendation of Bacteroidaceae and Butyrivibrio and descriptions of Desulfomonas gen. nov. and ten new species in the genera Desulfomonas, Butyrivibrio, Eubacterium, Clostridium, and Ruminococcus. Int J Syst Evol Microbiol Microbiol Soc.

[22] Nishiyama Y, Massey V, Takeda K, Kawasaki S, Sato J, Watanabe T (2001). Hydrogen peroxide-forming NADH oxidase belonging to the peroxiredoxin oxidoreductase family: existence and physiological role in bacteria. J Bacteriol.

[23] Masip L, Veeravalli K, Georgiou G (2006). The many faces of glutathione in bacteria. Antioxid Redox Signal.

[24] Kaitha S, Bashir M, Ali T (2015). Iron deficiency anemia in inflammatory bowel disease. World J Gastrointest Pathophysiol.

[25] Tailford LE, Owen CD, Walshaw J, Crost EH, Hardy-Goddard J, Le Gall G (2015). Discovery of intramolecular trans-sialidases in human gut microbiota suggests novel mechanisms of mucosal adaptation. Nat Commun.

[26] Dabard J, Bridonneau C, Phillipe C, Anglade P, Molle D, Nardi M (2001). Ruminococcin A, a new lantibiotic produced by a Ruminococcus gnavus strain isolated from human feces. Appl Environ Microbiol.

[27] Rigottier-Gois L (2013). Dysbiosis in inflammatory bowel diseases: the oxygen hypothesis. ISME J.

[28] Hansen SGK, Skov MN, Justesen US (2013). Two cases of Ruminococcus gnavus bacteremia associated with diverticulitis. J Clin Microbiol.

[29] Kim YJ, Kang HY, Han Y, Lee MS, Lee HJ (2017). A bloodstream infection by Ruminococcus gnavus in a patient with a gall bladder perforation. Anaerobe.

[30] Roux A-L, El Sayed F, Duffiet P, Bauer T, Heym B, Gaillard J-L (2015). Ruminococcus gnavus total hip arthroplasty infection in a 62-year-old man with ulcerative colitis. J Clin Microbiol.

[31] Fuentes S, Rossen NG, van der Spek MJ, Hartman JH, Huuskonen L, Korpela K (2017). Microbial shifts and signatures of long-term remission in ulcerative colitis after faecal microbiota transplantation. ISME J.

[32] Liu C, Finegold SM, Song Y, Lawson PA. Reclassification of Clostridium coccoides, Ruminococcus hansenii, Ruminococcus hydrogenotrophicus, Ruminococcus luti, Ruminococcus productus and Ruminococcus schinkii as Blautia coccoides gen. nov., comb. nov., Blautia hansenii comb. nov., Blautia hydrogenotrophica comb. nov., Blautia luti comb. nov., Blautia producta comb. nov., Blautia schinkii comb. nov. and description of Blautia wexlerae sp. nov., isolated from human faeces. Int J Syst Evol Microbiol. 2008;58:1896–902 10.1099/ijs.0.65208-0 18676476

[33] Lawson PA, Finegold SM (2015). Reclassification of Ruminococcus obeum as Blautia obeum comb. nov. Int J Syst Evol Microbiol.

