# Supplementary figures and images for: A novel Ruminococcus gnavus clade enriched in inflammatory bowel disease patients
Source: Genome Med. 2017 Nov 28;9:103. doi: 10.1186/s13073-017-0490-5 (PMC5704459; doi:10.1186/s13073-017-0490-5)

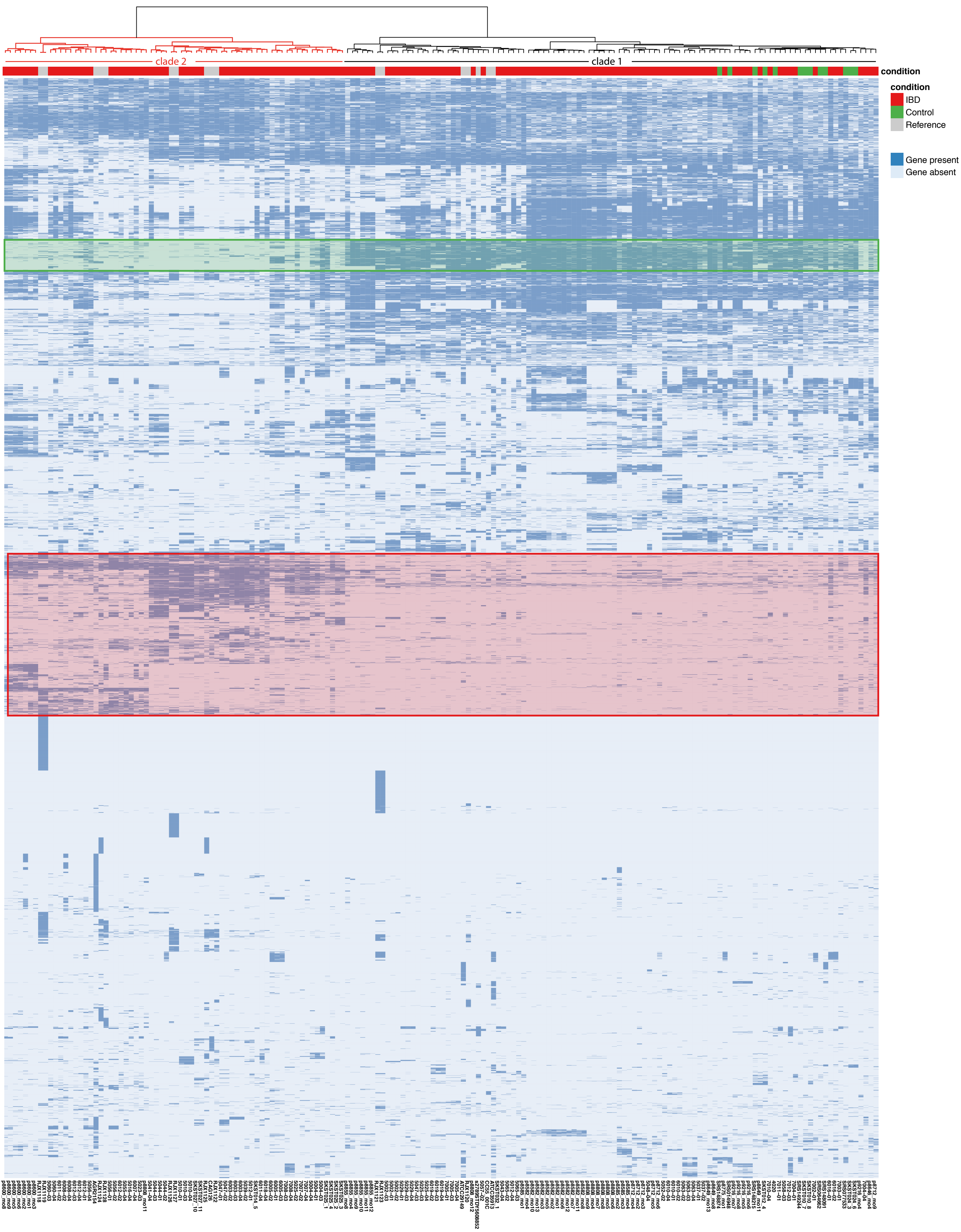

Supplement: Supplementary file 6 — Pangenome analysis on two distinct clades of R. gnavus. Pangenome analysis of R. gnavus using PanPhlAn showing the presence or absence of every gene in the R. gnavus pangenome. The x-axis shows metagenomic samples as well as R. gnavus reference genomes; the y-axis shows gene clusters from the R. gnavus pangenome. Clustering the results reveals two clades of R. gnavus, which we call clade 1 and clade 2. Using metagenomic samples from LSS/Lewis/HMP cohorts, we found that R. gnavus strains from all healthy adult controls and some IBD samples were functionally similar to R. gnavus clade 1. On the other hand, only IBD samples had R. gnavus strains which were functionally similar to R. gnavus clade 2. The red transparent rectangle contains genes enriched in R. gnavus group IBD. The green transparent rectangle contains genes many genes missing from R. gnavus group IBD. (PDF 2396 kb) [file 13073_2017_490_MOESM6_ESM.pdf]
